# Interatrial blocks prevalence and risk factors for human immunodeficiency virus-infected persons

**DOI:** 10.1371/journal.pone.0223777

**Published:** 2019-10-17

**Authors:** Francisco Fanjul, Antoni Campins, Javier Asensio, Gloria Sampériz, Aina Yañez, Dora Romaguera, Miquel Fiol, Melchor Riera

**Affiliations:** 1 Infectious Diseases Unit, University Hospital Son Espases, Palma de Mallorca, Spain; 2 Illes Balears Health Research Institute (IdISBa), Palma de Mallorca, Spain; 3 Infectious Diseases Unit, University Hospital Miguel Servet, Zaragoza, Spain; 4 Faculty of Nursing and Physiotherapy, Illes Balears University, Palma de Mallorca, Spain; 5 Physiopathology of Obesity and Nutrition CIBER (CIBER-OBN), Palma de Mallorca, Spain; Central University of Tamil Nadu, INDIA

## Abstract

**Background:**

Interatrial blocks are considered a new important risk factor for atrial fibrillation and cerebrovascular events. Their prevalence and clinical implications have been reported in general population and several subgroups of patients but no data from HIV-infected populations, with a non-negligible prevalence of atrial fibrillation, has been previously reported.

**Methods:**

We conducted a cross-sectional study in a previously enrolled cohort of randomly selected middle-aged HIV-infected patients who attended our hospital and were clinically stable.

Patients underwent both a 12-lead rest electrocardiogram and clinical questionnaires while epidemiological, clinical and HIV-related variables were obtained from electronic medical records and interviews with the patients. Electrocardiograms were then analyzed and codified using a standardized form by two trained members of the research team who were blinded to clinical variables.

**Results:**

We obtained electrocardiograms from 204 patients with a mean age of 55.22 years, 39 patients (19.12%) presented an interatrial block, 9 (4.41%) advanced and 30 (14.71%) partial. Patients with interatrial block had a lower nadir lymphocyte CD4 count (124 vs 198 cells, p = 0.02) while advanced interatrial blocks were associated to older age (62.16 vs. 54.95 years, p = 0.046) and hypertension (77.8% vs. 32.3%, p = 0.009). We did not find differences regarding baseline CD4 lymphocyte count or CD4/CD8 lymphocyte ratio. Clinical variables and functional capacity among patients with or without interatrial block were similar.

**Conclusions:**

In a cohort of clinically stable HIV infected patients the prevalence of interatrial blocks, specially advanced, is high and associated to previously known factors (age, hypertension) and novel ones (nadir CD4 lymphocyte count).

## Introduction

The prevalence of electrocardiographic abnormalities (aECG) among people living with HIV (PLWH) is higher than expected in the general population [1,2] and its presence has been found to be a good predictor of cardiovascular events [3]. Chronic HIV infection seems to have a deleterious effect over the cardiovascular system both directly through local viral replication and indirectly by causing accelerated aging in the context of maintained immune activation [4].

With regard to aECG, there are consistently reported data regarding prolonged QT interval [5], atrial fibrillation [6–8], left ventricular hypertrophy, and subclinical coronary disease [9] prevalence in PLWH from both clinical trials and cohort studies [3]; however, to our knowledge, no study has reported the prevalence of interatrial blocks (IAB) in PLWH. These blocks, described in 1979 by Bayés de Luna, are classified as partial (p wave duration ≥120 ms) or advanced (p wave duration ≥120 ms plus bifascicular morphology of p wave in II, III, and aVF, also known as Bayes Syndrome) [10] and are hypothesized to be the consequence of electric atrial remodeling and progressive dysfunction due to fibrosis [11, 12].

IABs have been consistently reported in the last decade as a key risk factor for atrial fibrillation and cardioembolic cerebrovascular events in the general population [13–15]. Their clinical relevance and implications, previously grossly underestimated, are still the subject of ongoing trials.

The prevalence of IAB in the general population depends mainly on age and it has been previously reported that at least 40% of the patients aged over 70 years present it [14,16]. However, the published data vary widely depending on the populations studied as IAB has been found to be more prevalent among patients with some comorbidities, such as obstructive sleep apnea [17], structural cardiopathy, or Chagas cardiomyopathy [18], among others.

Recent literature has reported that HIV infection could provoke delays in the interatrial conduction measured by echocardiography, which could result in a higher risk of IAB. Furthermore, these delays were associated with lower CD4 counts and the length of HIV infection so a relationship between immunity state and interatrial conduction was suggested [19].

Hypothesizing that the prevalence of IAB in PLWH would be high and probably related to chronic immunosenescence, we designed a study with the following aims: to report prevalence data on IAB; to analyze risk factors for IAB in a middle-aged population of PLWH, including nadir CD4 T lymphocyte count and CD4/CD8 ratio as traditional markers of immunosuppression and immunosenescence, respectively [20]; and to compare functional capacity and symptoms defined by clinical questionnaires and the 6-minute walk test between patients with or without an IAB.

## Materials and methods

### Design, settings, and participants

We conducted a single-center cross-sectional study in a sample of middle-aged PLWH. Patients were enrolled from a prospective cohort established in our center (Hospital Universitari Son Espases, Palma de Mallorca, Spain) between 2008–2010 that has been followed up since then. Detailed inclusion and exclusion criteria in that cohort have previously been published elsewhere [21]; briefly, a sample of 275 patients was randomly selected from those attending our hospital who met all the following criteria; a) age 40–69 years; b) clinical stability at admission; and c) signed informed consent. Patients were excluded if they presented with advanced heart, kidney, or liver disease, Karnofsky index <70, were pregnant, or had experienced an opportunistic infection in the prior month. They were also excluded if they were receiving systemic steroids, immunosuppressants, or chemotherapy at inclusion.

### Data collection

Between January 2014 and December 2016, all patients included in the initial cohort who were still followed up were offered the chance to complete clinical questionnaires (mMRC, St George Respiratory [22] and Rose Angina [23] questionnaires) and 12-lead resting ECG using the same ECG machine (TC-30, Philips, Amsterdam, Netherlands) with the low-pass filter at 100 Hz and the high-pass filter at 0.05 Hz. The obtained ECG was then codified by 2 different researchers who were blinded to the patient’s data using a standardized form to minimize bias. P wave duration was measured following current recommendations [24].

Clinical, epidemiological, and HIV infection-related variables, including nadir and baseline (obtained less than 3 months from the date of the ECG) CD4 counts, CD4/CD8 ratio, and HIV viral load, were obtained from electronic medical records and interviews with the patients.

We codified toxic habits in an ordinal way (current, former, or never) while comorbidities were registered on a presence/absence basis. Hepatitis C virus infection (HCV) was defined as detectable viremia or a positive test for the hepatitis C virus antibody. Chronic obstructive pulmonary disease (COPD) was defined according to international guidelines as a post-bronchodilator forced expiratory volume in 1 second (FEV1)/forced vital capacity (FVC) < 70% [25]. The prior cardiovascular disease variable was defined as the presence of any of the following: previous stroke, coronary ischemic event, pulmonary thromboembolism, and peripheral arterial disease.

### Statistical methods

We present categorical variables as percentages and continuous variables as the mean with standard deviation for normal distributions or median with interquartile range for non-normal distributions. Differences between patients with or without IAB were analyzed using Student’s t test for independent samples, analysis of variance (ANOVA), or Mann Whitney U for continuous variables as appropriate according to variable distribution. Categorical variable distribution between the 2 groups analyzed was compared using the chi square test or Poisson distribution as appropriate. Pre-specified subgroup analysis was planned for patients according to the type of IAB (advanced vs. partial vs. none).

We provide unadjusted odds ratios (OR) with 95% confidence intervals (CI) for analyzed variables. We also present adjusted ORs (95% CI) obtained from a multivariate binary logistic regression analysis that was performed using a stepwise approach. Predefined covariates for consideration due to their previously established relevance or their importance for this study objectives were: age, length of HIV infection, nadir CD4 count, and CD4/CD8 ratio. Variables with p values <0.10 in the univariate analysis were also considered for inclusion in the multivariate model. Covariates were tested for collinearity and a receiver operating characteristic (ROC) curve analysis was performed for the final model.

All tests were performed two-tailed when possible and a p value <0.05 was considered significant. All the statistical tests were performed with SPSS ver. 20 (IBM, Armonk, New York, USA).

### Ethics

The study was conducted in accordance with the Good Clinical Practice and ethical principles of the declaration of Helsinki. The protocol was reviewed and approved by the Ethics Committee of the Balearic Islands (IB2313/14PI). All participants signed their informed consent before undergoing any study procedure.

## Results

Two hundred and forty-five patients out of the 275 included in the initial cohort continued to attend our center in 2014. Among them, 207 agreed to participate in the cross-sectional study. Herein, we present data from 204 patients whose ECGs were considered technically acceptable. The mean patient age was 55.22 (SD: 6.72) years, 75.7% were men, and 97.05% were receiving highly active antiretroviral treatment. Only 7 patients presented with a baseline HIV viral load over >50 copies/ul and 69.7% of patients presented with a baseline CD4 count over 500 cells/mL. Table 1 shows the baseline characteristics of the patients.

**Table 1:** Baseline characteristics at inclusion.

*Variable*	*n = 204*
Sex (male)	75.7%
Age (y)	55.22+-6.72
BMI (kg/m2)	24.70+-4.37
Diabetes	12.4%
Hypertension	34.8%
DLP	61.7%
Previous CVD	12.4%
Familiar CVD	15.6%
COPD	24.7%
HCV	35.5%
Transmission IDUMSMOther	30.15%35.18%34.67%
SmokingCurrentFormerNever	50%30.5%19.5%
Pack-years	33.71+-21.17
HIV infection length	18.27+5.32
Baseline CD4 cells/μl (median)	657 (456–895)
Baseline CD4/CD8 (mean)	0.90 (0.64–1.29)
nadir CD4 cells/μl (median)	187 (75–281)
HIV Viral Load <50 copies/ul	96.5%
CDC StageABC	42.76%26.21%31.07%

BMI: Body mass index, DLP: dyslipidemia, CVD: Cardiovascular disease, COPD: Chronic obstructive pulmonary disease. HCV: Hepatitis C virus, IDU: Intravenous drug user, MSM: Men who have sex with men, CDC: Centers for disease control and prevention

The prevalence of traditional cardiovascular risk factors (diabetes, hypertension, hypercholesterolemia, and obesity) was high with 74.9% of the patients presenting with at least 1 factor and 11% presenting with 3 or more.

### Interatrial blocks

The mean p wave duration was 98.04 (SD: 15.06) ms and 39 (19.12%) patients presented with an IAB (9 advanced and 30 partial). Table 2 shows a comparison of variables among the 2 studied groups.

**Table 2:** Distribution of analyzed variables according to IAB presence.

*Variable*	IAB*n = 39*	No IAB*n = 165*	*p*
Sex (male)	87.2%	72.7%	0.07
Age (y)	55.81 (7.22)	55.14 (6.62)	0.58
BMI (kg/m2)	25.80 (4.08)	24.43 (4.41)	0.09
Diabetes	10.3%	12.7%	0.79
Hypertension	46.2%	31.5%	0.13
DLP	59%	61.2%	0.72
Previous CVD	2.6%	14.5%	0.05
Familiar CVD	15.4%	15.8%	1
COPD	20.5%	23.6%	0.54
HCV	33.3%	35.2%	0.85
Transmission IDUMSMOther	23.1%38.5%38.5%	30.9%33.3%32.7%	0.50
SmokingCurrentFormerNever	43.6%33.3%23.1%	50.3%29.1%18.2%	0.61
Pack-years	36.14 (21.52)	33.18 (21.26)	0.48
HIV infection length	16.82 (5.36)	18.56 (5.27)	0.07
Baseline CD4 cells/μl (median)	628 (443–850)	659 (461.5–905.8)	0.56
Baseline CD4/CD8 (mean)	0.94 (0.65–1.55)	0.89 (0.63–1.25)	0.81
nadir CD4 cells/μl (median)	124 (24–249)	198 (87.5–286.5)	0.02
CDC C stage	43.59%	28.49%	0.08
RCPQ (positive)	5.3%	3.2%	0.93
SGRQ	12.66 (14.50)	12.72 (15.04)	0.98
6MWT (% ref)	96.0 (10.5)	99.07 (26.3)	0.50

BMI: Body mass index, DLP: dyslipidemia, CVD: Cardiovascular disease, COPD: Chronic obstructive pulmonary disease. HCV: Hepatitis C virus, IDU: Intravenous drug user, MSM: Men who have sex with men, CDC: Centers for disease control and prevention, RCPQ: Rose Chest Pain Questionnaire, SGRQ: St. George’s Respiratory Questionnaire, 6MWT; 6-Minute Walking test

Patients who presented with an IAB had a lower nadir CD4 count (median: 124 cells/mL (IQR: 24–249) vs. 198 cells/mL (IQR: 87.5–286.5), p = 0.02). They also tended to be men and present a previous Centers for Disease Control (CDC) stage C disease; however, these differences did not reach statistical significance.

**Fig 1:**
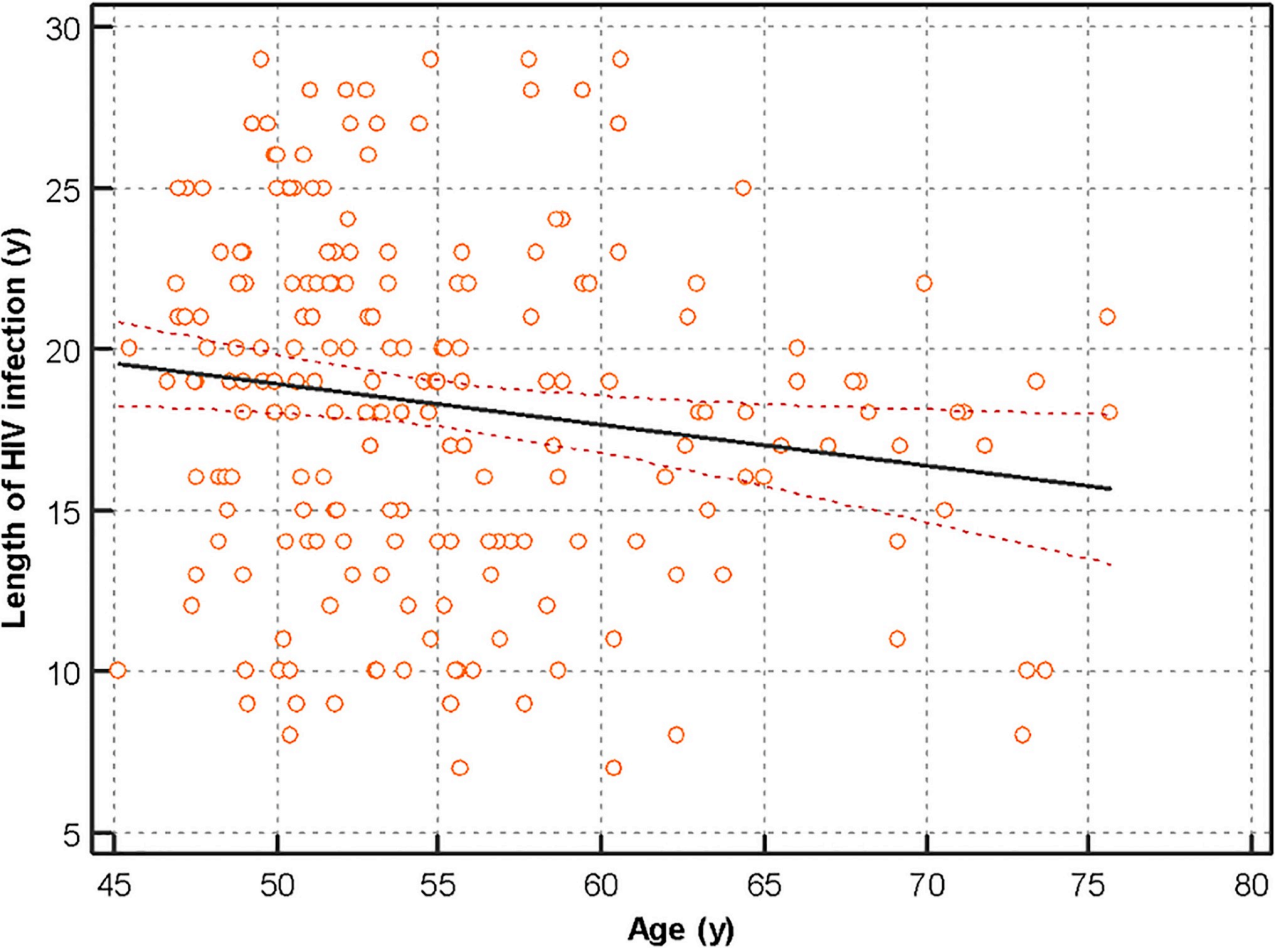
Correlation between length of HIV infection and patient´s age.

Patients did not differ significantly in smoking mechanism of transmission, or clinical performance in the included questionnaires and no significant differences in age between patients with or without IAB were present. Patients who presented with an IAB had been infected with HIV for a shorter time. Analyzing these results in bivariate analysis, we found that age and length of infection were inversely correlated in our sample, as shown in Fig 1. Complete risk factor analysis is shown in Table 3.

**Table 3:** Analysis of risk factors for IAB.

	Unadjusted OR	p	Adjusted OR	*p*
Sex (male)	2.55 (0.94–6.92)	0.07	3.02 (1.06–8.63)	0.039
Age (y)	1.01 (0.96–1.07)	0.573	1 (0.95–1.06)	0.992
BMI	1.07 (0.99–1.16)	0.09	1.08 (0.98–1.18)	0.106
Diabetes	0.77 (0.25–2.38)	0.65		
Hypertension	1.81 (0.89–3.69)	0.1		
DLP	0.87 (0.43–1.77)	0.70		
Obesity	1.18 (0.36–3.79)	0.79		
Previous CVD	0.15 (0.02–1.16)	0.07	0.13 (0.02–0.99)	0.049
Familiar CVD	0.97 (0.37–2.53)	0.94		
COPD	0.74 (0.31–1.75)	0.49		
HCV	0.89 (0.42–1.86)	0.75		
Smoking (ever)	0.76 (0.33–1.78)	0.53		
HIV infection length (y)	0.94 (0.88–1.01)	0.07	0.96 (0.89–1.03)	0.244
Nadir CD4 cells/μl			0.997 (0.99–1)	0.025
Nadir CD4 <100 cells/μl	1.97 (0.95–4.08)	0.07		
Nadir CD4 <200 cells/μl	1.76 (0.857–3.63)	0.12		
CDC C stage	1.94 (0.95–3.98)	0.07		

BMI: Body mass index, DLP: dyslipidemia, CVD: Cardiovascular disease, COPD: Chronic obstructive pulmonary disease. HCV: Hepatitis C virus, CDC: Centers for disease control and prevention.

**Fig 2:**
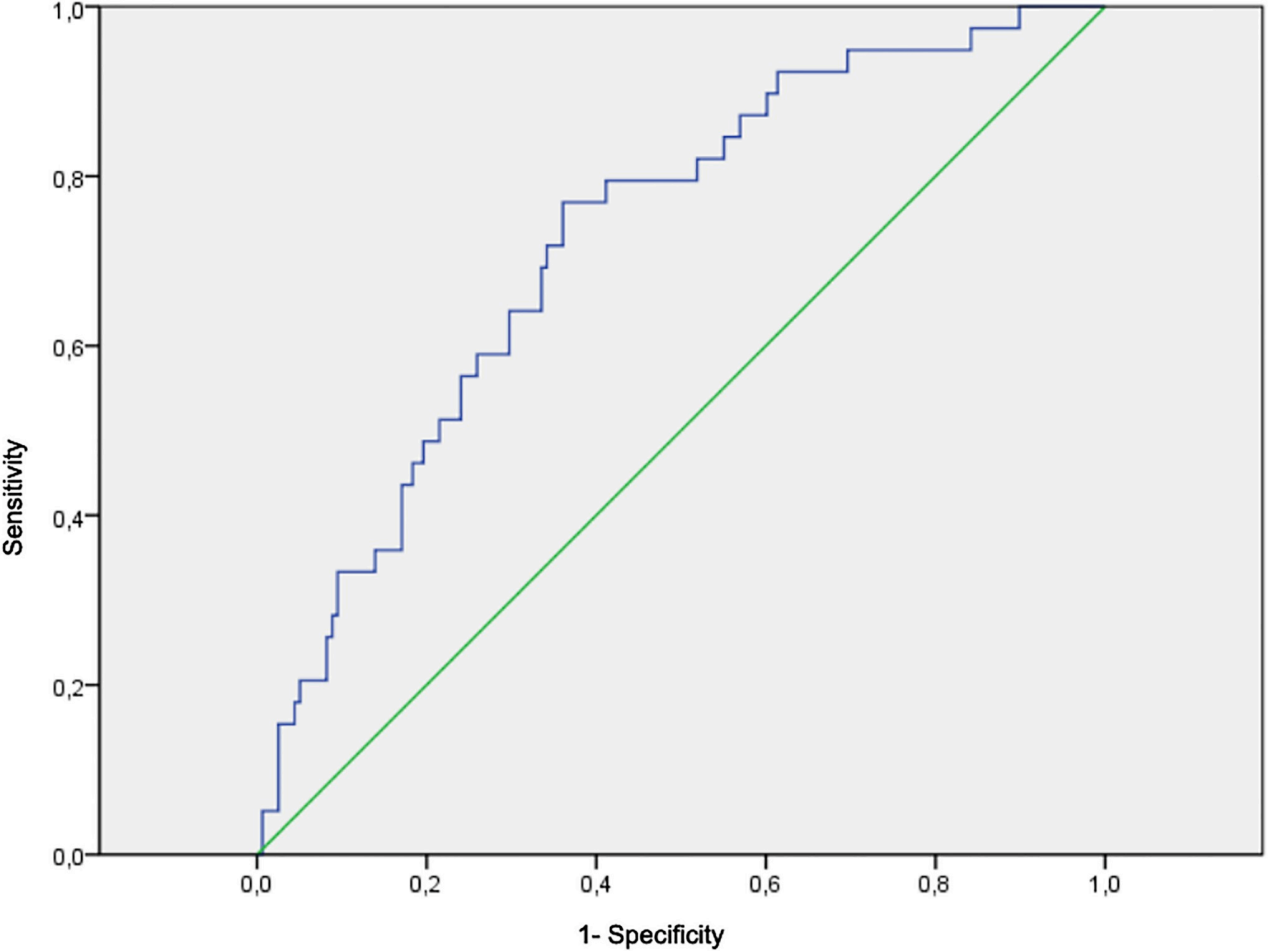
ROC curve of the final model. Nagelkerke r2: 0.16. ROC AUC: 0.726 (0.642–0.811) P<0.001.

The final multivariate model included covariates of age, sex, length of HIV infection, CD4 nadir, body mass index, and previous cardiovascular disease. Fig 2 shows the ROC curve for the combined probabilities with an area under the curve of 0.726 (0.642–0.811) P<0.001.

### Advanced interatrial blocks

**Fig 3:**
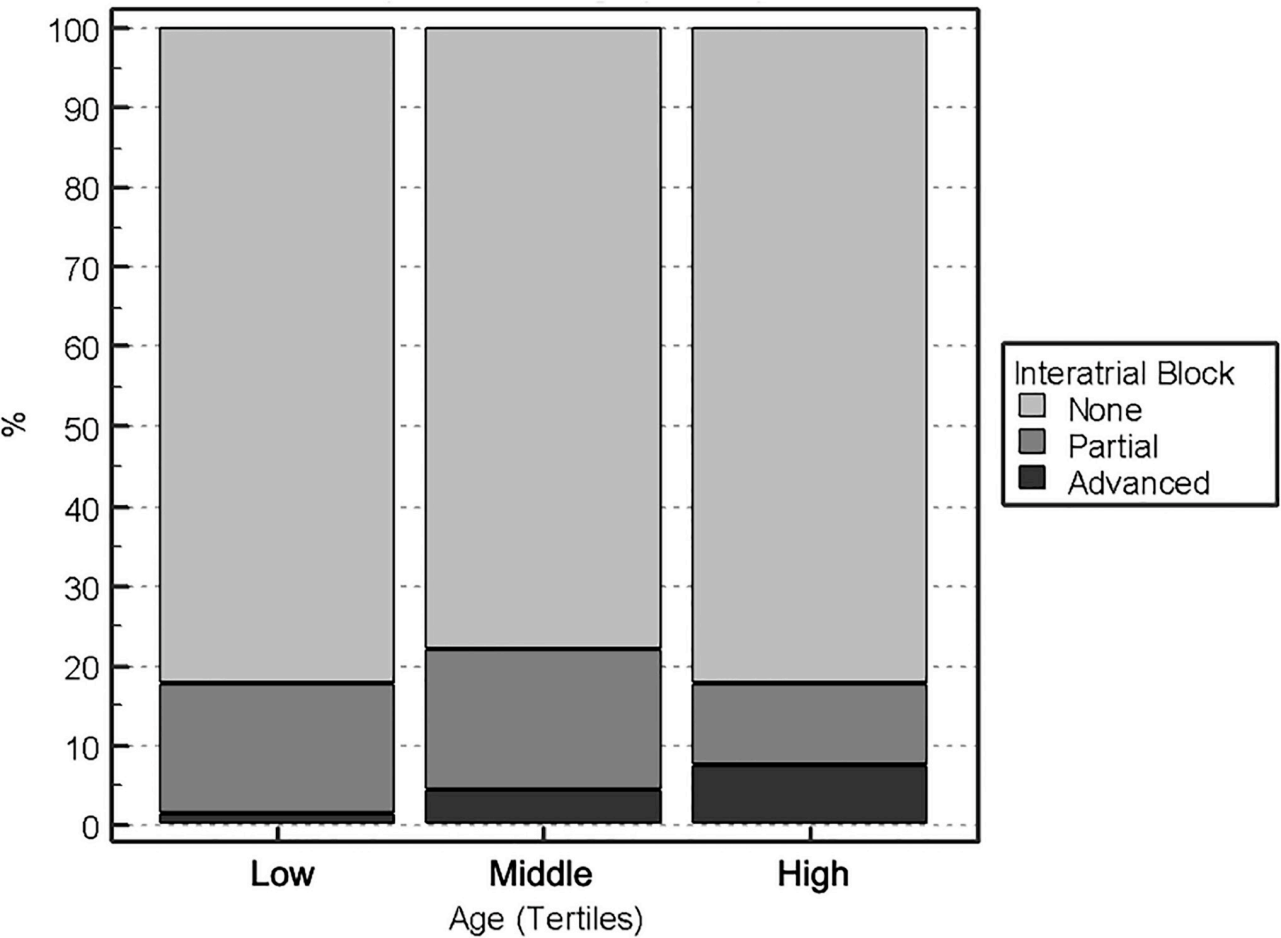
Prevalence of IAB according to age.

Patients with advanced interatrial blocks (aIAB) were significant older than those without (62.16 (SD: 9.11) vs. 54.95 (SD: 6.45) years, p = 0.046) and presented with hypertension more frequently (77.8% vs. 32.3%, p = 0.009). Fig 3 depicts the present trend among age tertiles in aIAB while Table 4 shows the characteristics of the patients according to aIAB and unadjusted ORs for the main variables. We did not attempt to perform multivariable analysis because of the small sample.

**Table 4:** Baseline characteristics according to aIAB.

*Variable*	aIAB*n = 9*	No aIAB195	p	Unadjusted OR (CI95%)	p
**Sex (male)**	88.9%	74.9%	0.458	2.69 (0.33–22.01)	0.358
**Age (y)**	62.16 (9.11)	54.95 (6.45)	0.046	1.13 (1.04–1.13)	0.004
**BMI**	25.56 (4.85)	24.75 (4.18)	0.570		
**Diabetes**	11.1%	12.3%	1		
**Hypertension**	77.8%	32.3%	0.009	7.17 (1.45–35.5)	0.016
**DLP**	77.8%	60%	0.487		
**Previous CVD**	0%	12.8%	0.605		
**Familiar CVD**	22.2%	15.4%	0.635		
**COPD**	33.3%	22.6%	0.692		
**HCV**	22.2%	35.4%	0.496		
**Transmission** IDUMSMOther	11.1%33.3%55.6%	30.3%34.3%32.8%	0.454		
**Smoking** CurrentFormerNever	33.3%55.6%11.1%	49.7%28.7%19.5%	0.385		
**Pack-years**	35.39 (23.22)	33.61 (21.24)	0.808		
**HIV infection length**	16.56 (4.21)	18.30 (5.36)	0.338	0.94 (0.83–1.07)	0.339
**Baseline CD4 cells/μl**	564 (424.5–868)	659 (455.5–895.75)	0.886		
**Baseline CD4/CD8 mean**	1.08 (0.55–1.93)	0.89 (0.64–1.26)	0.803		
**Nadir CD4 cells/μl**	137 (56.5–246)	187 (75–283)	0.125	1 (0.99–1.00)	0.601
**CDC C stage**	44.4%	30.8%	0.466		
**SGRQ**	11.86 (15.92)	12.75 (14.89)	0.862		
**6MWT (% ref)**	94.33 (16.34)	98.59 (24.28)	0.671		

BMI: Body mass index, DLP: dyslipidemia, CVD: Cardiovascular disease, COPD: Chronic obstructive pulmonary disease. HCV: Hepatitis C virus, IDU: Intravenous drug user, MSM: Men who have sex with men, CDC: Centers for disease control and prevention, SGRQ: St. George’s Respiratory Questionnaire, 6MWT; 6-Minute Walking test

### Other electrocardiographic abnormalities

The prevalence of specific aECGs is presented in Table 5. Most of the analyzed aECGs had a prevalence lower than 5–10%; however, all aECGs were considered, 83.3% of the patients included presented with at least one of the reported abnormalities in the ECG.

**Table 5:** Prevalence of electrocardiographic findings.

*Electrocardiographic findings(n = 204)*	
**Beats per minute** (lpm, media ± DE)	68.63 ± 10.69
**Sinus bradycardia**	14.5%
**Sinus rhythm**	85.5%
**Atrial fibrillation or flutter**	0%
**PR interval duration** (ms, media ± DE)	158.16 ± 26.62
**PR >200ms**	6.1%
**QRS duration** (ms, media ± DE)	89.76 ± 15.88
**QRS > = 110ms**	12.5%
**Pathological Q wave**	6%
**Left ventricular hypertrophy**	9.1%
**QTc interval duration** (ms, media ± DE)	394.02 ± 54.74
**Prolonged QTc**	5.5%
**Left bundle branch block**	2.03%
**Left bundle branch hemiblock**	2.03%
**Right bundle branch block**	1.52%
**Any ST segment abnormality**	52.3%
**Significant ST segment abnormality**	40.1%
**Any T wave abnormality**	60.4%
**Atrial extrasystoles**	0.5%
**Ventricular extrasystoles**	2.6%

## Discussion

We report the first prevalence data of IAB in an HIV-infected population. Almost 20% of the included patients presented with an IAB (14.71% partial and 4.41% advanced) and its presence was related to age, sex, hypertension, and nadir CD4.

To our knowledge, there are no previous studies in HIV with which to compare our prevalence results. Comparing our findings to studies performed in general population [14,16], our prevalence of global IAB is similar or even lower than that previously published. In contrast, the prevalence of aIAB is higher in our study than previously described. O’Neal et al. reported data from more than 14,000 patients of a similar age (54±5.8 years vs. 55.22±6.72 years) and found an aIAB prevalence of 0.5% and an incident rate of 2.27 cases/1,000 person-years.

We can only hypothesize about the causes of this difference but it seems reasonable to consider that chronic HIV infection could probably increase the risk of aIAB because of inflammation leading to subsequent fibrosis. This theory could be supported by previous evidence regarding delayed interatrial conduction in PLWH [19] and the observed relationship between nadir CD4 and IAB, which has not been previously reported. Age, male sex, and hypertension had been previously found to be risk factors for IAB by O´Neal et al.; therefore, our results are consistent with previous literature.

The clinical relevance of this study is yet to be determined. In the last few years, increasing evidence about the impact of IAB on clinical outcomes, including hospital admissions, poor functional capacity, and mortality has been published [26]; however, until now, there are no recommendations for the follow-up or treatment of these patients so it remains to be seen how identifying patients with IAB could benefit their health. In the case of PLWH (who are considered at higher cardiovascular risk than uninfected people), the global rapid increase in mortality due to cardiovascular events requires a more proactive approach in implementing prevention strategies that could aim for lessening the burden of disease.

### Our study has some limitations

The characteristics of our sample did not allow us to determine if the prevalence of IAB was related to the length of HIV infection as this variable was inversely correlated with age in our sample. Furthermore, our sample is relatively small and our study does not have the statistical power to identify all the risk factors involved in the prevalence of IAB. Also, the lack of a control group of HIV uninfected patients made impossible for us to directly assess the impact of HIV infection in the incidence of IAB. In order to limit this, we have resorted to compare our results with those previously published.

In conclusion, aIAB seem to be more prevalent than expected among PLWH and is related to both previously known (age, sex, hypertension) and novel (nadir CD4) variable. Therefore, more studies should aim to address and confirm IAB prevalence, identify IAB risk factors, and develop strategies aimed to reduce its associated risk of cardiovascular events in PLWH.
